# Transcriptional and Epigenetic Landscape of Cardiac Pacemaker Cells: Insights Into Cellular Specialization in the Sinoatrial Node

**DOI:** 10.3389/fphys.2021.712666

**Published:** 2021-07-16

**Authors:** Ravi Mandla, Catherine Jung, Vasanth Vedantham

**Affiliations:** Division of Cardiology, Department of Medicine and Cardiovascular Research Institute, University of California, San Francisco, San Francisco, CA, United States

**Keywords:** sinoatrial node, cardiac pacemaker cell, transcriptional regulation, enhancer, ATAC-seq and chromatin accessibility, heart rate, sinus node dysfunction

## Abstract

Cardiac pacemaker cells differentiate and functionally specialize early in embryonic development through activation of critical gene regulatory networks. In general, cellular specification and differentiation require that combinations of cell type-specific transcriptional regulators activate expression of key effector genes by binding to DNA regulatory elements including enhancers and promoters. However, because genomic DNA is tightly packaged by histones that must be covalently modified in order to render DNA regulatory elements and promoters accessible for transcription, the process of development and differentiation is intimately connected to the epigenetic regulation of chromatin accessibility. Although the difficulty of obtaining sufficient quantities of pure populations of pacemaker cells has limited progress in this field, the advent of low-input genomic technologies has the potential to catalyze a rapid growth of knowledge in this important area. The goal of this review is to outline the key transcriptional networks that control pacemaker cell development, with particular attention to our emerging understanding of how chromatin accessibility is modified and regulated during pacemaker cell differentiation. In addition, we will discuss the relevance of these findings to adult sinus node function, sinus node diseases, and origins of genetic variation in heart rhythm. Lastly, we will outline the current challenges facing this field and promising directions for future investigation.

## Cardiac Pacemaker Cells Are Specialized Cardiomyocytes With a Distinct Gene Expression Program

**FIGURE 1:**
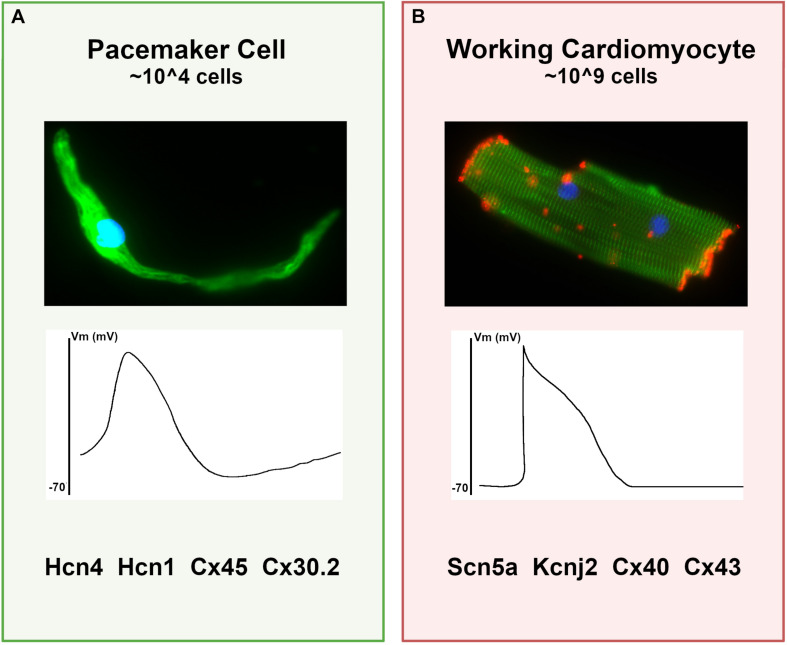
Differences between pacemaker cells and working cardiomyocytes. **(A)** Characteristics of typical pacemaker cells: (top panel) micrograph of an isolated pacemaker cell taken from an adult *Hcn4-GFP* mouse demonstrates an elongated spindle-like morphology; (middle panel) a typical pacemaker cell action potential with elevated resting membrane potential and diastolic depolarization; (bottom panel), a list of key functional proteins enriched in pacemaker cells. **(B)** For comparison, typical ventricular cardiomyocyte characteristics are shown, including (top panel) a micrograph of an adult mouse ventricular cardiomyocyte stained for alpha-actinin (green) to demonstrate sarcomeres and connexin-43 (red) to demonstrate gap junctions; (middle panel) a typical working cardiomyocyte is quiescent with a negative resting potential, rapid upstroke, and plateau phase; (bottom panel) key functional genes expressed in working cardiomyocytes that are reduced in pacemaker cells.

Pacemaker cells comprise a small population of specialized cardiomyocytes within the sinoatrial node (SAN) that spontaneously fire to trigger each heartbeat. Although pacemaker cells are contractile and have sarcomeres, they possess striking phenotypic differences from atrial cardiomyocytes, including smaller cell size with elongated morphology, cellular protrusions, and robust electrical automaticity with weak intercellular electrotonic coupling (Figure 1). In the context of the SAN, these cellular specializations allow pacemaker cells to perform their primary function – dynamic impulse generation at rates that ensure cardiac output meets metabolic need.

Over the past several decades, a prevailing model for the cellular basis of pacemaker cell function has emerged in which mutual entrainment of a membrane clock (driven by hyperpolarization-activated cation current) and a calcium clock (driven by spontaneous sub-sarcolemmal calcium release) confers robust automatic firing (Lakatta et al., 2010). In parallel, investigations at the tissue level have shown that the SAN has a distributed architecture that spans the region from the superior vena cava-right atrium junction to the inferior vena cava, bounded by the crista terminalis and the interatrial septum (Boyett et al., 2000). Nodal tissue consists of a fibrous matrix in which centrally located pacemaker cells are poorly electrotonically coupled with the atrial myocardium while pacemaker cells at the SAN periphery, where impulse transmission occurs, are more robustly coupled to the atrium (Joyner and van Capelle, 1986; De Maziere et al., 1992; Nikolaidou et al., 2012). This architecture allows for source-sink matching between the SAN and atrium without compromising automaticity.

Depending on neurohormonal state and electrolyte concentration, the nodal impulse can arise from different locations within the intercaval groove, generating an electrical wavefront that ultimately exits the SAN through species-specific pathways (Nikolaidou et al., 2012). Aligned with this conceptual framework, recent evidence has demonstrated the existence of discrete nodal structures within the SAN region that control heart rate under different conditions, with a superior SAN (located at the superior vena cava-RA junction) containing the dominant pacemaker at rapid heart rates and an inferior SAN (located near the inferior vena cava) operating at lower rates (Brennan et al., 2020). Finally, the atrioventricular node, which can function as a subsidiary pacemaker, also contains pacemaker-like cells that exhibit automaticity and hyperpolarization-activated cation current.

Remarkably, despite the anatomical and physiological complexity in heart rate regulation, both the cellular physiology of pacemaker cells and the tissue architecture in the SAN are broadly similar among diverse mammalian species despite dramatic variation in body mass and heart rate, suggesting that the determinants of cardiac automaticity constitute a robust and deeply evolutionarily conserved morphogenetic and cellular program (Bleeker et al., 1980; Opthof et al., 1985). In keeping with this notion, many of the specific molecular components required for pacemaker cell automaticity in the SAN have been identified and are present in pacemaker cells isolated from widely divergent vertebrate species (fish, mouse, and human), including hyperpolarization-activated, cyclic-nucleotide gated ion channels (Hcn4, Hcn1) and lower conductance gap junctions (connexin-45, connexin 30.2) (Blaschke et al., 2007; Tessadori et al., 2012). Conversely, high-conductance gap junctions such as connexin-43 and connexin-40, as well as channels associated with rapid conduction (NaV1.5) and hyperpolarized resting potential (Kir2.1), are present at much lower levels in pacemaker cells than in atrial cardiomyocytes (Mommersteeg et al., 2007b). More recently, genome wide expression profiles using RNA sequencing on mouse and human SAN have identified numerous common transcripts that are differentially expressed between SAN and non-SAN cardiomyocytes in both species, indicative of a shared transcriptional program that underlies the phenotypic similarities among mammalian pacemaker cells in different species (Figure 1; Vedantham et al., 2015; Goodyer et al., 2019; van Eif et al., 2019).

It is thus clear that (1) the problem of generating a robust mechanism for heart rate regulation was solved early in vertebrate evolution through the differentiation, proliferation, and architectural assembly of a specialized subtype of cardiomyocyte at the venous pole of the heart; and (2) pacemaker cells become functionally specialized by executing a conserved gene expression program with increased transcription of genes responsible for spontaneous firing and autonomic connectivity, and decreased transcription of genes associated with force generation and rapid conduction.

By connecting cellular specialization at the phenotypic level to cell type-specific gene expression programs, this unified model for the origin of vertebrate heart rhythm raises several important questions that will be the focus of the present article. First, what are the upstream regulators that allow pacemaker cells to specialize and differentiate during heart development? Second, how do these regulators turn on the SAN gene program and how is this program maintained? Third, what is the relevance of these regulatory pathways to human genetic variation and human disease? Historically, our ability to address these questions has been limited by the lack of genetic tools to mark and track pacemaker cells in model systems, as well as the difficulty of isolating sufficient quantities of purified pacemaker cells for molecular analysis. Therefore, in the present work, we will also touch on technological breakthroughs in the past decade that have allowed us to define gene expression and chromatin dynamics in pacemaker cells with unprecedented genomic resolution.

## A Distinct Set of Transcription Factors Is Enriched in Pacemaker Cells and Is Required to Establish a Specialized Gene Program

In general, tissue-specific gene expression is accomplished through cell-type specific transcription factors (TFs) that bind combinatorially and often synergistically to *cis*-acting genomic DNA sequences known as enhancers that can be located far from their target genes (more on this below). These binding events, in turn, permit the formation of transcriptional complexes between TF-bound enhancers and basal promoters of target genes to activate expression. The specific complement of activated transcriptional regulators available to bind genomic DNA has been termed the “nuclear regulatory environment.” The nuclear regulatory environment in a given cell, in combination with the distribution of available enhancers, determines which genes are expressed.

Pacemaker cells express a set of transcriptional regulators that are absent in atrial cardiomyocytes: Tbx3, Tbx18, Shox2, and Isl1 (Hoogaars et al., 2004; Christoffels et al., 2006; Blaschke et al., 2007; Sun et al., 2007; Weinberger et al., 2012). These factors could therefore function as cell-intrinsic regulators that directly activate gene expression of pacemaker cell-specific genes and/or repress expression of the atrial gene program. Conversely, there are transcriptional regulators that are present in atrial cardiomyocytes that are excluded from pacemaker cells, including Nkx2.5 and Pitx2c, that may be important for activating atrial genes and repressing pacemaker cell genes (Christoffels et al., 2006; Mommersteeg et al., 2007b). Over the past 15 years, several rigorous *in vivo* global and conditional loss of function studies have been carried out in animal models with each of these factors, leading to a general model for transcriptional control summarized in this section.

### Repression of the Atrial Gene Program in Pacemaker Cells

Several pacemaker cell-enriched transcription factors directly repress expression of atrial-enriched genes in the SAN. By blocking differentiation toward a working cardiomyocyte cell fate, this inhibition is hypothesized to permit differentiation along the path to pacemaker cells.

*Tbx3*, a T-box transcription factor whose expression within the SAN is conserved across several species, is the most well-characterized factor to play this role. Tbx3 binds to the regulatory elements that control expression of working myocardial genes such as *Nppa* and *Gja5*, possibly competing with Tbx5 (an activating T-box factor) to inhibit their expression and thereby inhibit chamber differentiation within the SAN (Hoogaars et al., 2004). Mice lacking Tbx3 demonstrate ectopic expression of atrial genes within the SAN with SAN hypoplasia, and exhibit embryonic lethality, whereas conditional deletion of Tbx3 within the adult SAN also results in ectopic atrial gene expression in the SAN and sinus arrhythmias (Hoogaars et al., 2007). Underscoring its primary role as a repressor, transgenic misexpression of *Tbx3* within atrial myocardium can repress the atrial gene program and even result in some ectopic arrhythmogenic foci, but it does not directly activate expression of pacemaker cell genes ectopically.

Analogous to Tbx3, Tbx18 also appears to function as a repressor of atrial genes such as Gja5 (Wiese et al., 2009; Kapoor et al., 2011). However, as the central sinus node fails to form in the Tbx18^–/–^ mouse, additional specific targets of Tbx18 within the SAN have not been defined using loss of function models (Christoffels et al., 2006). In a large animal model, Tbx18 can reprogram ventricular myocardium to exhibit automaticity and transiently pace the heart, although the transcriptional mechanisms underlying this effect have not been determined (Kapoor et al., 2011, 2013).

The homeodomain transcription factor Shox2 also restricts atrial gene expression within pacemaker cells to promote SAN development. Complete loss of Shox2 causes embryonic lethality with hypoplasia of the sinus venosus, reduced embryonic heart rate, and ectopic expression of atrial genes in the SAN domain (Blaschke et al., 2007; Espinoza-Lewis et al., 2009). Recent findings have suggested a competitive mechanism whereby Shox2 directly represses Nkx2.5 expression in pacemaker cells and occupies sites normally bound by Nkx2.5 in atrial myocardium to repress atrial gene expression, thereby maintaining indirectly the expression of genes such as Hcn4, Tbx3, and Isl1 within the SAN (Ye et al., 2015). In myocardium at the interface between the SAN and the atrium, where Shox2 and Nkx2.5 are both expressed at higher levels, this competition may produce an intermediate cellular phenotype that facilitates electrophysiological source-sink matching between the SAN interior and the atrium (Li et al., 2019).

### Repression of Pacemaker Cell Program in Atrial Cardiomyocytes

Within the working myocardium, the cardiac TF Nkx2.5 may act to repress expression of pacemaker cell genes. For example, conditional deletion of Nkx2.5 in ventricular myocardium results in upregulation of Hcn1, a gene normally restricted to pacemaker cells (Pashmforoush et al., 2004). Further supporting such a role, Nkx2.5 is among the few core cardiac TFs that is downregulated in pacemaker cells and their embryonic precursors in the sinus venosus (Christoffels et al., 2006). Transgenic misexpression of Nkx2.5 in pacemaker cells leads to repression of both Hcn4 and Tbx3, key components of the pacemaker cell-specific gene regulatory network (Espinoza-Lewis et al., 2011).

The homeodomain transcription factor Pitx2c is expressed throughout the left atrial and pulmonary vein myocardium, where it is largely responsible for encoding left sided identity within the atrium (Liu et al., 2002; Mommersteeg et al., 2007a). Accordingly, Pitx2c^–/–^ mice can develop right atrial isomerism and form a secondary SAN in the left atrium that is normally repressed by the presence of Pitx2c (Mommersteeg et al., 2007b). Importantly, the Pitx2 locus harbors genomic variation that contributes to inherited susceptibility to atrial fibrillation, an arrhythmia that is triggered by focal firing from the region of the pulmonary venous myocardium and left-sided SAN remnant (Roselli et al., 2018; Zhang et al., 2019).

### Activation of Specialized Gene Expression Programs in Pacemaker Cells and Atrial Myocardium

One of the major remaining unknowns in pacemaker cell transcriptional regulation is how pacemaker cell-specific gene expression is activated in the SAN. Data from avians and zebrafish have suggested that pacemaker cell progenitors arise from mesodermal cells located at the periphery of the embryonic heart fields in response to a Wnt signal (Bressan et al., 2013; Ren et al., 2019), and subsequently adopt their unique phenotype and transcriptional program as they become integrated with the atrial myocardium in a dynamic cellular process that occurs after heart looping (Bressan et al., 2018; Thomas et al., 2021).

Once committed, pacemaker cells express the broad cardiac transcription factors Gata4, Gata6, Mef2C, and Tbx5 at relatively high levels during development, similar to atrial cardiomyocytes (Vedantham et al., 2015; van Eif et al., 2019). These factors cooperatively activate the cardiac gene expression program, and their binding sites are overrepresented near both atrial cardiomyocyte and pacemaker cell genes (Galang et al., 2020; van Eif et al., 2020). Accordingly, depletion of any of these factors profoundly affects SAN development, heart rhythm, and heart development more broadly (Molkentin et al., 1997; Bruneau et al., 2001; Moskowitz et al., 2007; Kuratomi et al., 2009). Thus, although these factors are undoubtedly relevant to directing pacemaker cell-specific genes to the SAN, they do not fully account for the cell type-specificity of expression seen in pacemaker cells. One possibility is that within pacemaker cells, the repressive TFs Tbx3 and Shox2 bind to loci important for working myocardium and thereby cause Mef2C, Gata4, and Tbx5 to redistribute occupancy to pacemaker cell-specific loci where they activate transcription. This mechanism would not necessarily require any additional pacemaker cell-specific factors to explain pacemaker cell-specific gene expression.

More recently, the LIM homeodomain TF Isl1 has emerged as a potential activator of pacemaker cell-specific gene expression that could explain how pacemaker cells differentiate along a transcriptional path that is distinct from the atrial cardiomyocyte program. Although Isl1 is expressed broadly in cardiac progenitor cells of the second heart field, its expression becomes restricted to the SAN and its precursors by mid-development onward (Cai et al., 2003; Sun et al., 2007). Conditional deletion of Isl1 within the SAN results in dysregulated gene expression with major downregulation of pacemaker cell-specific genes and some upregulation of atrial genes (Liang et al., 2015). Moreover, these Isl1 SAN-conditional loss of function embryos exhibit sinus bradycardia and SAN hypoplasia, confirming a critical role for this factor in function and morphogenesis of the SAN. Analysis of promoter-adjacent regions of SAN genes downregulated after deletion of Isl1 demonstrated enrichment of Isl1 binding sites, a finding that raises the likelihood of an activating role for Isl1 in pacemaker cell-specific gene expression (Vedantham et al., 2015).

## Regulatory Basis for Cellular Specialization: Role of Genome Architecture and Chromatin Accessibility

While the genetic models discussed in the previous section have provided insight into the factors that likely activate and repress cell-type specific gene expression patterns in pacemaker cells, the mechanisms that connect these factors to their target genes have not been elucidated. To a great extent, this knowledge gap has resulted from the inability to identify specific genomic loci (enhancers) where combinations of pacemaker cell and other TFs bind directly and regulate transcription of specific genes. In the absence of such direct binding information, the connection between TFs and their targets cannot be definitively established, and a common logic to combinatorial activation of pacemaker cell gene expression cannot be defined. Thus, identifying enhancers that exhibit pacemaker cell-specific activity would add another crucial piece to this complex puzzle. Before reviewing recent experiments that have tackled this problem, this section briefly outlines our current model for how enhancers and promoters interact with each other in the context of the nucleus, and the experimental methods that can be used to identify enhancers.

### Organization of Genomic DNA in the Nucleus Facilitates Transcription

**FIGURE 2:**
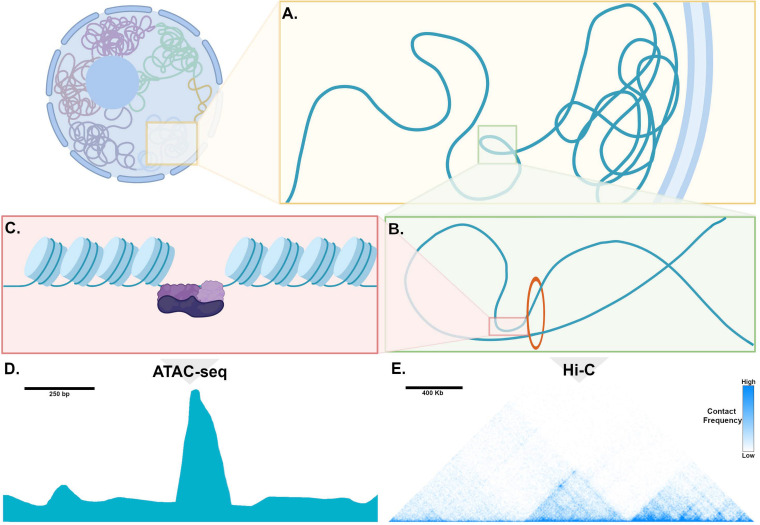
Genome organization, chromatin, and mechanisms of transcription. **(A)** Within the nucleus, chromosomes occupy distinct territories and within a given territory, compacted, non-transcribed DNA is clustered into lamina-associated domains (LADs) adjacent to the nuclear periphery. DNA that is actively transcribed is located in the interior of the nucleus, where is it organized into topologically associated domains (TADs). **(B)** Enhancers and promoters are brought into proximity through loop extrusion by the ring-like Cohesion complex bound to *Ctcf* (red circle). **(C)** Chromatin is tightly wound around histone complexes. At sites of active enhancers, histone proteins are modified, allowing access of transcription factors (purple) to bind to DNA. **(D)** An example of processed ATAC-seq data aligned with the cartoon in **(C)**, expressed as a graph of read count versus genomic location, showing a “peak” or region of open chromatin, where histones are modified to make DNA accessible to a transposase. **(E)** An example of processed Hi-C data expressed as a heatmap, where the horizontal axis reflects genomic location. Pixel color represents the number of read counts, corresponding to the extent of contact frequency in *cis* between two regions of genomic DNA positioned along a chromosome. Topologically associated domains, visualized as discrete triangular shaped structures on the heatmap, are defined by regions that are permissive for long-range contacts.

In mammalian cells, chromosomes each have distinct territories within the nucleus, and within each territory, transcriptionally inactive genomic loci are sequestered at the nuclear periphery, while actively transcribed regions are positioned in the interior of the nucleus (Figure 2A; van Steensel and Belmont, 2017). These transcriptionally active chromatin regions are organized into topologically associated domains (TADs), which can range in length from kilobases to megabases and can contain one or many genes along with non-coding regions that contain enhancers (Figure 2B; Dixon et al., 2016). TADs are formed when the multifunctional cohesin protein complex is recruited by chromatin-bound CCCTC-binding factor (CTCF). Cohesin then extrudes chromatin through the center of its ring-like structure to form a loop. Loop extrusion occurs until Cohesin encounters CTCF bound in the inverted orientation, thereby fixing the boundaries of the TAD along the genome and defining the regions for potential interaction. Importantly, during the process of loop extrusion, distant chromatin elements along the TAD are brought into close proximity, permitting contact of TF-bound enhancers with target promoters, and thereby initiating transcription (Figure 2B; Hanssen et al., 2017). Genomic regions such as enhancers and promoters within a TAD can interact whereas interactions across TAD boundaries rarely occur.

In order for DNA binding TFs to access enhancers, histone proteins undergo covalent modifications at specific sites that relax their otherwise tight association with genomic DNA, which then assumes an “open chromatin” conformation (Figure 2C; Boland et al., 2014). These histone modifications are regulated by specific classes of enzymes, and in some cases, by a group of TFs known as “pioneer factors” that can bind to their sites in nucleosomal DNA in a “closed configuration” and recruit factors that result in opening of chromatin and binding of larger transcriptional complexes (Larson et al., 2021). In this manner, enhancers can “read out” the nuclear regulatory environment to determine whether a specific gene should be transcribed. Thus, in order to understand how transcription factors regulate their target genes in differentiated cell types, comparing the chromatin landscape among closely related cell types can uncover the specific set of enhancers that activate tissue-specific gene expression.

### Experimental Approaches to Epigenetic Profiling: Relevance to Pacemaker Cells

Because chromatin at transcriptionally active enhancers must be accessible to DNA binding factors, the Assay for Transposase Accessible Chromatin with Sequencing (ATAC-seq) offers a conceptually straightforward method to identify regulatory elements genome-wide. In this technique, a bacterial Tn5 transposase is used to insert oligonucleotide tags into regions of accessible chromatin (locations where histones are in the relaxed or open conformation). Sequencing these regions in a given cell type allows for high-resolution determination of regions of genome accessibility. ATAC-seq datasets can be compared among closely related cell types to identify loci of differential accessibility that might contain tissue-specific enhancers (Figure 2D; Buenrostro et al., 2013). ATAC-seq can be performed successfully on tens of thousands of cells and more recently, single cell ATAC-seq methodologies are available (Buenrostro et al., 2015), making this a practical tool for use with cardiac pacemaker cells directly isolated from SAN tissue.

Several factors complicate the interpretation of ATAC-seq data: First, differentially accessible genome regions are not always transcriptionally active enhancers; Second, a single enhancer can have more than one gene target; Third, because most TADs contain multiple genes, it is not always clear how to assign a putative enhancer to one or more specific gene targets within its TAD. One possible experimental approach to address these problem is to define contact frequencies among distant genomic loci through chromosome conformation capture and its variants (Sati and Cavalli, 2017). By covalently crosslinking genomic DNA and then sequencing crosslinked fragments, libraries can be generated in which sequences include fragments from an enhancer and the distant promoter it regulates since these will be in close spatial proximity during active transcription. With enough library complexity and sequencing depth, a genome-wide proximity map at tens of kilobase resolution can be readily generated to define mutually interacting regions within each TAD (Figure 2E). Variants of this approach, including promoter-capture Hi-C, can enrich libraries for promoter-containing fragments, thereby compiling a high-fidelity list of promoter -enhancer interactions in a given cell type (Mifsud et al., 2015; Schoenfelder et al., 2015; Montefiori et al., 2018).

Unfortunately, the limiting number of pacemaker cells (∼10,000 per heart) presents an insurmountable barrier for chromosome conformation capture, which ordinarily requires 10–20 million cells. In the future, scalable *in vitro* strategies to differentiate pacemaker cells from induced pluripotent stem cells may provide enough material, or, alternatively, lower input chromosome conformation techniques may be become available. Until then, connecting putative enhancers in pacemaker cells with target genes will require experimental validation using *in vivo* models.

## Chromatin Accessibility Profiling of Pacemaker Cells Connects Transcription Factors to Their Target Genes

**FIGURE 3:**
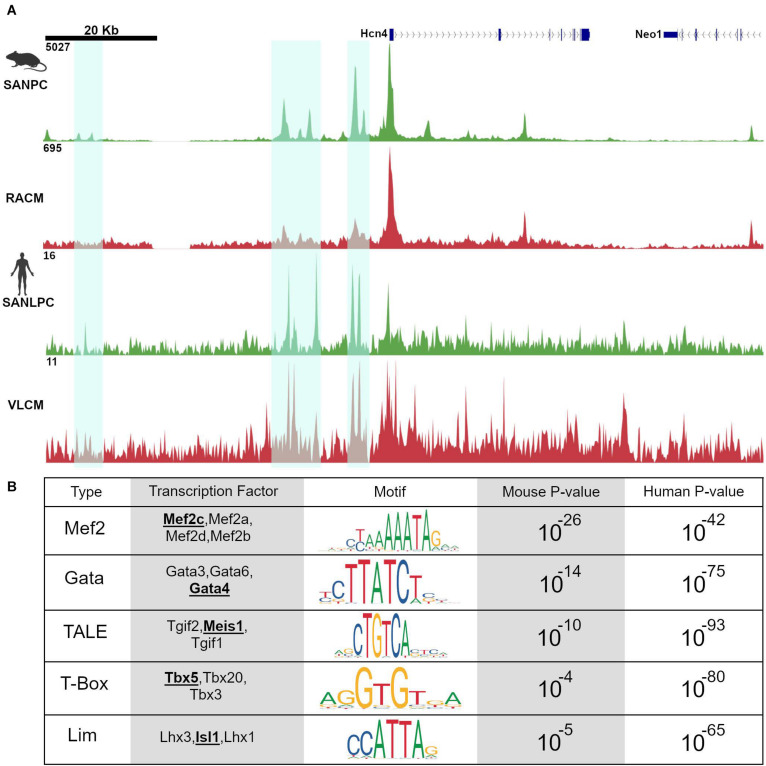
Pacemaker cell-specific chromatin accessibility profiles are deeply conserved. **(A)** Alignment of ATAC-seq data at the *Hcn4* genomic locus from mouse neonatal pacemaker cells with human induced pacemaker-like cells demonstrate regions of open chromatin that are present in both species, highlighted in cyan. **(B)** Motif enrichment analysis of differentially accessible regions between SAN and non-SAN myocytes demonstrated that enrichment of similar transcription factor motifs are present in mouse and human pacemaker cells, suggesting a conserved mammalian pacemaker cell transcriptional program. SANLPC, sinoatrial node pacemaker cells; RACM, right atrial cardiomyocytes; SANLPC, sinoatrial node-like pacemaker cells; VLCM, ventricular-like cardiomyocytes.

Recently, several ATAC-seq data sets derived from pacemaker cells in mouse and human model systems have provided important new information about regulatory networks in pacemaker cells. Using the Hcn4-GFP transgenic mouse, both (Fernandez-Perez et al., 2019) and (Galang et al., 2020) used ATAC-seq to profile accessible chromatin regions in pacemaker cells (Fernandez-Perez et al., 2019; Galang et al., 2020). Fernandez Perez et al. compared these results to accessibility profiles of murine embryonic fibroblasts reprogrammed into induced pacemaker-like cells, while Galang et al. compared them to accessibility profiles from right atrial cardiomyocytes, a closely related cell type to pacemaker cells. In a parallel study, van Eif et al. (2020) used ATAC-seq to identify pacemaker-specific accessible chromatin regions in human cells. By implementing a recently described protocol for deriving pacemaker-like cells from induced pluripotent stem cells, van Eif et al. compared accessible chromatin regions between induced pacemaker-like cells (SANLPC) and induced ventricular-like cardiomyocytes (VLCM). As detailed below, these three datasets exhibited a remarkable congruence and have thereby provided novel insights into a deeply conserved *cis*-regulatory architecture in pacemaker cells (Figure 3A).

### Motif Enrichment Analysis and *in vivo* Transgenic Assays Demonstrate a Deeply Conserved Regulatory Code in Pacemaker Cells

Because regions of accessible chromatin are bound by tissue-specific transcription factors, binding sites for lineage-determining TFs should be overrepresented in chromatin regions that are differentially accessible between closely related cell types. Accordingly, both Galang et al. and van Eif et al. looked at DNA binding motifs enriched in differentially accessible regions as compared to background sequences using an unbiased search algorithm. The results revealed a striking convergence in the motifs in both sets of differentially accessible regions. Unsurprisingly, known cardiomyocyte transcription factor motifs including Gata and T-box motifs were overrepresented. More strikingly, differentially accessible regions from both data sets, drawn from very different types of samples (mouse primary pacemaker cells versus human iPSC-derived SANLPC), showed overrepresentation of Isl1 TF binding motifs, as well as binding motifs for Meis1, a TALE-class homeodomain factor not previously studied in the context of SAN gene expression (Figure 3B). Similar findings were observed in the data set from Fernandez-Perez et al. (2019). The finding of overrepresentation of Isl1 motifs among the differentially accessible peaks in pacemaker cells, in particular, provides strong evidence that Isl1 is a transcriptional activator in pacemaker cells and that, together with interacting partners, is likely to be a key player in activating the genes that endow pacemaker cells with their unique phenotypes.

To test whether differentially accessible ATAC-seq peaks were sufficient to function as regulatory elements *in vivo*, both Galang et al. and van Eif et al. cloned selected differentially accessible ATAC-seq peaks they identified and tested them using enhancer-reporter transgenesis in mice and in zebrafish. In Galang et al., 17 differentially accessible peaks were selected and tested using EnSERT, a locus-specific transgenesis assay, or using an *hsp68LacZ* reporter relying on random insertion. Of these, 4 ATAC-seq peaks directed reporter activity to the SAN primordium in mouse embryos; notably, these peaks were located within TADs that encompassed *Hcn4*, *Rgs6*, *Ptgfr*, and *Isl1*, all of which are differentially expressed in pacemaker cells. The enhancer at the *Isl1* locus, in particular, exhibited remarkable specificity for pacemaker cells in the developing and postnatal heart, suggesting that it functions within the pacemaker cell lineage from a very early developmental stage onward.

In a similar vein, van Eif et al. tested 8 human differentially accessible ATAC-seq peaks located within TADs encompassing SHOX2, ISL1, and TBX3, and identified several with expression in the heart and sinus venosus in zebrafish, of which 3 had activity in the sinus venosus region of mice (2 at the SHOX2 locus and one at the TBX3 locus). Notably, the human region syntenic to the Isl1 enhancer identified in mouse pacemaker cells by Galang et al. was also strongly differentially accessible in the human ATAC-seq data from van Eif et al. providing a convincing example of cross-species conservation of genomic regulatory architecture. Indeed, both studies found that enhancer function was conserved across species to a remarkable extent – human sequences were active in mouse and fish, and mouse sequences were active in fish – often with the expected tissue-specific expression pattern. The fact that the nuclear regulatory environment in zebrafish pacemaker cells can “read out” both human and mouse DNA to achieve tissue-specific expression indicates that the regulatory kernel involving Isl1, Shox2, and other pacemaker cell TFs is an ancient vertebrate regulatory module that has been conserved over hundreds of millions of years.

### Enhancer Knockouts Demonstrate Required Roles for Enhancers in Gene Regulation, Development, and Function

**FIGURE 4:**
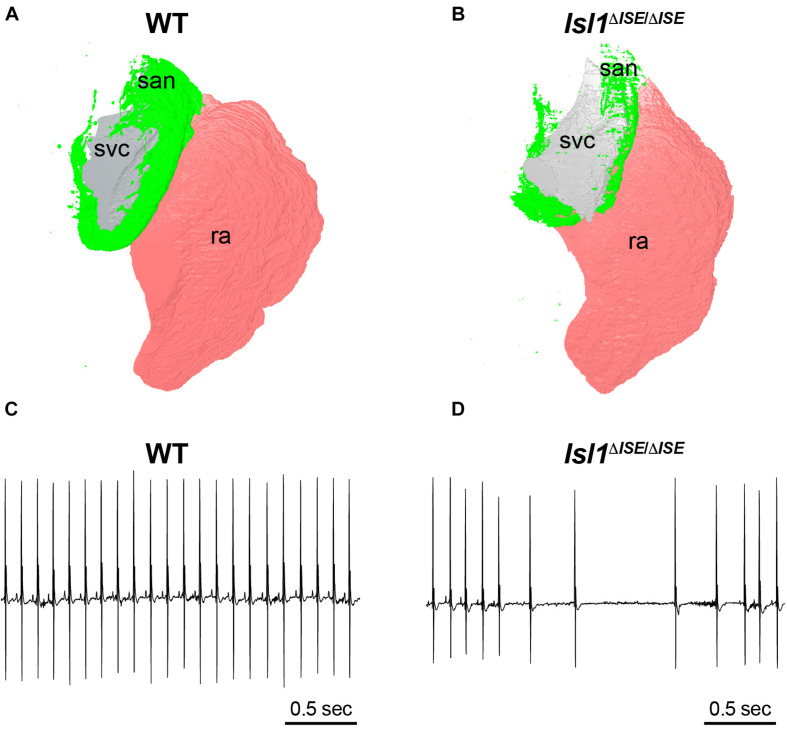
Sinoatrial node hypoplasia and dysfunction after deletion of a key pacemaker cell-specific enhancer. **(A)** Three-dimensional reconstruction of light sheet fluorescence image of *Hcn4* + sinoatrial node (san, green), right atrium (pink, ra) and superior vena cava (gray, svc) from a WT control adult heart (left) in comparison to **(B)** a heart obtained from a littermate lacking both copies of an SAN-specific enhancer (ISE) for the transcription factor *Isl1*. Hearts were stained in whole mount with Hcn4 antibody and san, ra, and svc were manually segmented. Note the markedly reduced size of the Hcn4 + SAN in the *Isl1^Δ^^*ISE/*Δ^^*ISE*^* heart. **(C)** Electrocardiograms recorded from a WT control adult mouse (left) and **(D)** an *Isl1^Δ^^*ISE/*Δ^^*ISE*^* littermate demonstrate an example of sinus arrhythmias in animals lacking the Isl1 enhancer.

To test the functional relevance of these enhancers, both Galang et al. and van Eif et al. deleted regulatory sequences from the genome of mice to test whether target gene expression and SAN development are affected. In Galang et al., a 2.7-Kb segment at the Isl1 locus (hereafter, *Isl1*-Locus SAN Enhancer, or *ISE*) was deleted using CRISPR-Cas9. Homozygous *Isl1^Δ*ISE/*Δ*ISE*^* mice were born in Mendelian ratios and were viable and fertile with no major cardiac structural abnormality by echocardiography. However, detailed molecular and histological studies showed that Isl1 protein and mRNA expression were reduced specifically in *Isl1^Δ*ISE/*Δ*ISE*^* pacemaker cells, with a corresponding hypoplasia of the SAN and reduced pacemaker cell number (Figures 4A,B). Furthermore, heart rhythm monitoring revealed a slower heart rate in the mutant mice, as well as episodes of sinus arrhythmia and bradycardia, indicative of abnormal SAN function (Figures 4C,D). Taken together, these findings support a model in which *ISE* supports *Isl1* transcription during SAN morphogenesis to ensure that the SAN achieves its normal size and cellularity, allowing for normal SAN function.

van Eif et al. deleted larger genomic regions within the SHOX2 and TBX3 TADs that contained differentially accessible ATAC-seq peaks. Examination of knockout embryos from a 250-kb deletion mouse line at the *Shox2* locus showed loss of Shox2 expression in the SAN, with dysregulation of Shox2 target genes, hypoplasia of the SAN, and embryonic lethality presumed due to bradycardia. In a parallel experiment, a 280-kb putative regulatory region containing differentially accessible ATAC peaks was also deleted from the Tbx3 locus. Homozygous mutant mice had absent Tbx3 expression in the SAN and exhibited perinatal lethality, while heterozygous mice survived to adulthood and exhibited prolonged SAN recovery times with programmed stimulation, indicating an important role for this enhancer in regulating SAN electrophysiology.

### Building a Model for Transcriptional Regulation in Pacemaker Cells Based on Loss of Function Studies and Epigenetic Profiling

**FIGURE 5:**
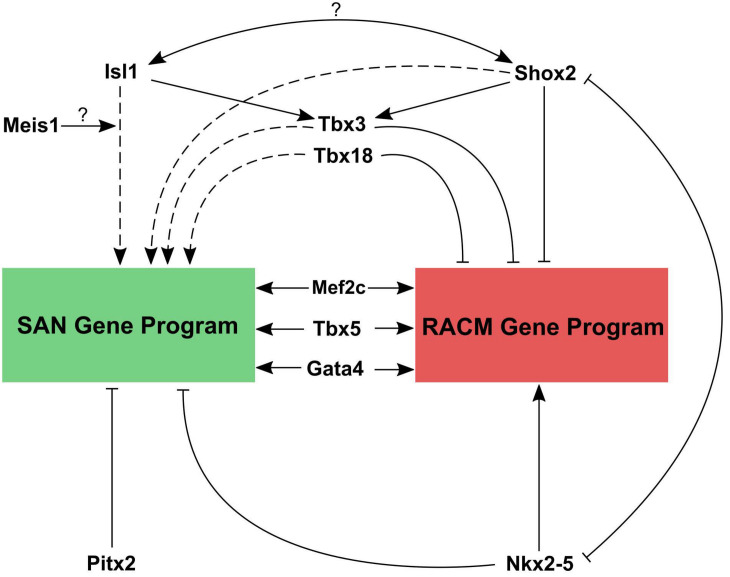
Working model for transcriptional regulation in pacemaker cells. Transcriptional control of the SAN gene program and right atrial cardiomyocyte gene program are contrasted. Both tissues require the cardiac factors Mef2, Gata4, and Tbx5 to active cardiac gene expression. However, reciprocal repression of SAN genes by Nkx2.5 and atrial genes by Tbx3, Tbx18, and Shox2, along with activation of SAN genes by Isl1 (with possible interacting partners) allows for cell type specific gene expression patterns. Question marks indicate a hypothesized interaction that has not been tested yet. Solid lines represent direct promotion/inhibition and dashed lines represent either direct or indirect promotion/inhibition.

By combining data from conditional loss of function studies for critical TFs, available ChIP-seq datasets for these factors, and the insights gleaned from the recent epigenetic profiling studies, a working model for transcriptional regulation in pacemaker cells can be constructed (Figure 5). Based on loss of function studies, Shox2 and Isl1 act upstream of Tbx3, which downregulates the atrial gene program. Shox2 also represses Nkx2.5 and its target genes. As noted above, within SAN pacemaker cell-specific ATAC-seq peaks, Isl1 binding motifs are enriched, suggesting it acts primarily as an activator of the SAN gene program. Outside of the SAN, Nkx-2.5 and Pitx2c repress the differentiation of additional pacemaker cells, further localizing the primary pacemaker of the heart specifically to the SAN. Overlapping ChIP-seq data for Gata4, Mef2c, and Tbx5 with SAN-specific ATAC-seq peaks confirms the binding of these TFs to putative SAN enhancers as well as more broadly to cardiac enhancers (Galang et al., 2020). *In vivo* studies have also demonstrated Mef2’s upstream role in regulating both SAN and non-SAN genes, similar to Tbx5 and Gata4 (Vedantham et al., 2013). Remaining questions include the identities of factors that may cooperate with Isl1 to regulate transcription in pacemaker cells. Since Isl1 is broadly expressed in other non-cardiac tissues, it is unlikely that Isl1 alone is sufficient to activate the pacemaker cell gene program. Candidate genes include Shox2 and Meis1, whose motifs are overrepresented near Isl1 sites in pacemaker cell-specific enhancers, as well as core cardiac TFs such as Tbx5, Gata4, Mef2, and others. In the coming years, as low-input ChIP-seq methodologies such as CUT&TAG are deployed more broadly, answers to these questions will be forthcoming.

## Relationship of Gene Regulation in Pacemaker Cells to Human Variation and Disease

Ultimately, a major goal of dissecting regulatory networks in pacemaker cells is to gain a better understanding of the pathophysiology of sinus node dysfunction and, more generally, to provide insight into the genetic underpinnings of variation in human heart rhythm. A number of previous studies have related genetic variation in heart rhythm parameters to regions of non-coding DNA that were subsequently found to contain enhancer that regulate expression of nearby genes (Christophersen et al., 2017; Roselli et al., 2018). Indeed, genome wide association studies (GWAS) have identified numerous single nucleotide polymorphisms (SNPs) in non-coding DNA that confer susceptibility to atrial fibrillation. Most notably, the chromosome 4q25 genomic locus, which exhibits the highest association with atrial fibrillation, contains enhancers that control expression of the homeodomain transcriptional factor Pitx2, a key regulator of left atrial identity and pulmonary venous myocardium that also inhibits growth of the left-sided SAN node primordium (Roselli et al., 2018).

**FIGURE 6:**
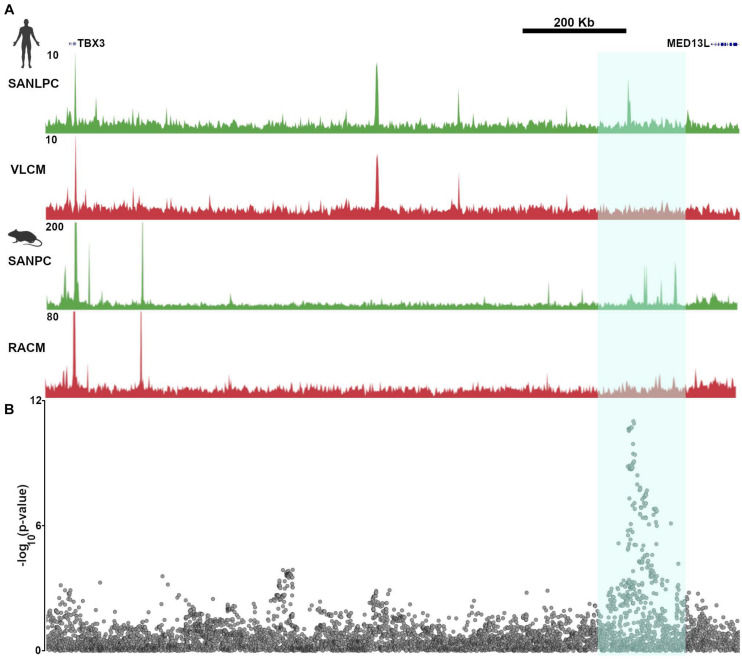
Common genetic variants in pacemaker cell enhancers affect sinoatrial node function in human populations. **(A)** Aligned SAN ATAC-seq data from human iPSC-derived pacemaker-like cells (SANLPC) and ventricular like cells (VLCM) with syntenic genomic region from mouse sinoatrial node pacemaker cells (SANPC) and right atrial cardiomyocytes (RACM) demonstrate differentially accessible chromatin regions shared by both sets of pacemaker cells (highlighted in cyan). **(B)** shows alignment of a resting heart rate GWAS at the TBX3 locus, with highly significant associations seen in the region with differentially accessible ATAC-seq peaks, underscoring the relevance of SAN enhancers to human sinoatrial node function. GWAS results were downloaded from http://www.nealelab.is/uk-biobank/.

In keeping with these findings, GWAS that have been performed using resting heart rate or heart rate recovery after exercise have found numerous “hotspots” in non-coding regions of the genome that were presumed to have regulatory function (Kilpeläinen, 2016; Ramirez et al., 2018). One such region is closest to the gene MED13L, but also lies within a TAD that encompasses TBX3. SNPs in this region display strong associations with heart rate recovery after exercise. Not surprisingly, the GWAS hot spot overlaps all of the *TBX3* enhancers that were identified and validated in van Eif et al. (2020), providing a conclusive demonstration of the relevance of epigenetic profiling to human heart rhythm (Figure 6). In the absence of the ATAC-seq data derived from pacemaker cells and the subsequent confirmatory studies, the association of a gene desert near MED13L with heart rate recovery would remain merely speculative.

Galang et al. worked from the presumption that ATAC-seq peaks were more likely than other regions of the genome to harbor heart rate-associated SNPs, prompting them to perform a new association study on UK Biobank participants, limiting the analysis to SNPs occurring within 500 bp on either side of the human genomic regions syntenic to mouse pacemaker cell ATAC-seq peaks. The results of this analysis demonstrated many associations between heart rate and SNPs occurring near ATAC-seq peaks that are close to established genes that regulate heart rate. Furthermore, by focusing the association study on a subset of SNPs, and hence reducing the number of hypotheses tested, the uncorrected *p* value threshold needed to establish a significant association was reduced. Thus, by focusing only on differentially accessible peaks that are open in pacemaker cells, Galang et al. (2020) uncovered a significant association between heart rate and SNPs in the vicinity of the Isl1 SAN enhancer described above. Although the effect size was small, alongside the rest of the association study, this finding establishes that enhancers are important genomic loci for determination of sinus node function, and that epigenetic profiling techniques such as ATAC-seq have the potential to illuminate the genetic basis for variation in heart rhythm at the population level.

## Outstanding Questions and Future Directions

The data summarized in this article have brought us closer than ever before to a detailed understanding of how pacemaker cells acquire their unique phenotypes during development, and to connecting the genetic determinants of pacemaker cell biology to vertebrate evolution and human physiology. Nevertheless, critical knowledge gaps remain.

First, while ATAC-seq data establish the genomic locations of the regulatory elements important for pacemaker cell-specific gene expression and indicate some of the transcription factors that are likely to bind to them, further work is needed to establish the precise timing and location of transcription factor binding events onto these enhancers. For example, which enhancers are specifically bound by Isl1 and what are its binding partners? What are the pioneer factors that open chromatin to allow for execution of the pacemaker cell gene expression program and what are the signaling pathways that activate these factors during sinus node development? Are these same pathways required for maintenance of pacemaker cell identify in the adult heart? How is epigenetic and transcriptional control related to age-related SAN dysfunction? Answers to these questions will fill in the missing pieces in our model for transcriptional control in pacemaker cells and will point the way toward translational strategies that could reverse SAN failure or even regenerate pacemaker cells.

A second area that will require further exploration is the connection between human genetic variation and SAN disease. As discussed, genome-wide and more limited association studies have demonstrated convincingly that a significant portion of human variation in SAN behavior is attributable to genetic differences at enhancer loci that regulate expression levels of critical SAN genes. A remaining challenge will be to take these analyses a step further by exploring whether variation in human non-coding regions is associated with the development of age-related or premature-onset sinus node dysfunction, atrial arrhythmias, and need for pacemaker implant. Defining the nature of these associations and the regulatory pathways involved could lead to new targets for interventions to prevent or treat SAN disease and other atrial arrhythmias.

Historically, research on these aspects of pacemaker cell and SAN biology has been challenging because of the difficulty of isolating pacemaker cells in large numbers, the lack of a suitable *in vitro* system to model pacemaker cells, and the limited access to human primary pacemaker cells. Fortunately, new tools and approaches are rapidly becoming available to surmount these technical hurdles. First, the development of human iPSC-based *in vitro* differentiation protocols, as highlighted in the study by van Eif et al., allows for the possibility of exploring the functions of *cis*-regulatory elements and transcriptional regulators in a more realistic human context without the limits imposed by low cell numbers. Thus, techniques such as ChIP-seq and Hi-C will eventually be used to create detailed, functionally annotated genomic maps of pacemaker cells. These datasets can then be integrated with data emerging from increasingly large cohorts of genotyped patients, including those with whole-genome sequencing alongside clinical data, to provide us with a detailed high-resolution model for how the epigenome and transcriptional biology connect with disease.

Alongside these technical innovations, the development of single cell genomic profiling technologies, including scRNA-seq and scATAC-seq, will circumvent the need to purify primary pacemaker cells for molecular analysis. Furthermore, data using these techniques can now be integrated across different platforms, allowing for more powerful *in silico* examination of cellular subtypes and cellular differentiation. Used in combination with new technologies that can isolate individual nuclei from differentiated tissues, these techniques will be invaluable as individual genes and pathways are explored in loss- and gain-of-function animal models and in small samples derived from human tissue. Taken together, the combination of recent technical and scientific advances leaves the field poised for rapid progress in the next several years.

## Conclusion

Over the last several decades, an integrated model for the electrophysiological basis of pacemaker cell automaticity and SAN physiology has taken shape, but a detailed understanding of the underlying gene regulatory networks has remained elusive. Recent advances in developmental biology, chromatin biology, bioinformatics, and human genetics have now revealed that several key transcriptional regulators and genomic loci are critical for pacemaker cell development, SAN formation, and SAN function, and have clarified their relevance to human variation. Work in the coming years will translate these findings to an improved understanding of SAN disease, novel targets for therapeutics, and possibly SAN regeneration.

## Author Contributions

RM and VV wrote the manuscript and prepared the figures. CJ prepared the figures, performed the imaging and image processing, and edited the text for intellectual content. All authors contributed to the article and approved the submitted version.

## Conflict of Interest

VV received research support from Amgen and a consulting fee from Merck for an unrelated project. The remaining authors declare that the research was conducted in the absence of any commercial or financial relationships that could be construed as a potential conflict of interest.

## Abbreviations

ATAC-seq: assay for transposase accessible chromatin
SAN: sinoatrial node.

## References

[B1] Blaschke R. J. Hahurij N. D. Kuijper S. Just S. Wisse L. J. Deissler K. (2007). Targeted mutation reveals essential functions of the homeodomain transcription factor Shox2 in sinoatrial and pacemaking development. *Circulation* 115 1830–1838. 10.1161/circulationaha.106.637819 17372176

[B2] Bleeker W. K. Mackaay A. J. Masson-Pevet M. Bouman L. N. Becker A. E. (1980). Functional and morphological organization of the rabbit sinus node. *Circ. Res.* 46 11–22. 10.1161/01.res.46.1.11 7349910

[B3] Boland M. J. Nazor K. L. Loring J. F. (2014). Epigenetic regulation of pluripotency and differentiation. *Circ. Res.* 115 311–324. 10.1161/circresaha.115.301517 24989490 PMC4229506

[B4] Boyett M. R. Honjo H. Kodama I. (2000). The sinoatrial node, a heterogeneous pacemaker structure. *Cardiovasc. Res.* 47 658–687. 10.1016/s0008-6363(00)00135-8 10974216

[B5] Brennan J. A. Chen Q. Gams A. Dyavanapalli J. Mendelowitz D. Peng W. (2020). Evidence of superior and inferior sinoatrial nodes in the mammalian heart. *JACC Clin. Electrophysiol.* 6 1827–1840. 10.1016/j.jacep.2020.09.012 33357580 PMC7770336

[B6] Bressan M. Henley T. Louie J. D. Liu G. Christodoulou D. Bai X. (2018). Dynamic cellular integration drives functional assembly of the heart’s pacemaker complex. *Cell Rep.* 23 2283–2291. 10.1016/j.celrep.2018.04.075 29791840 PMC6007983

[B7] Bressan M. Liu G. Mikawa T. (2013). Early mesodermal cues assign avian cardiac pacemaker fate potential in a tertiary heart field. *Science* 340 744–748. 10.1126/science.1232877 23519212 PMC3651765

[B8] Bruneau B. G. Nemer G. Schmitt J. P. Charron F. Robitaille L. Caron S. (2001). A murine model of Holt-Oram syndrome defines roles of the T-box transcription factor Tbx5 in cardiogenesis and disease. *Cell* 106 709–721. 10.1016/s0092-8674(01)00493-7 11572777

[B9] Buenrostro J. D. Giresi P. G. Zaba L. C. Chang H. Y. Greenleaf W. J. (2013). Transposition of native chromatin for fast and sensitive epigenomic profiling of open chromatin, DNA-binding proteins and nucleosome position. *Nat. Methods* 10 1213–1218. 10.1038/nmeth.2688 24097267 PMC3959825

[B10] Buenrostro J. D. Wu B. Litzenburger U. M. Ruff D. Gonzales M. L. Snyder M. P. (2015). Single-cell chromatin accessibility reveals principles of regulatory variation. *Nature* 523 486–490. 10.1038/nature14590 26083756 PMC4685948

[B11] Cai C. L. Liang X. Shi Y. Chu P. H. Pfaff S. L. Chen J. (2003). Isl1 identifies a cardiac progenitor population that proliferates prior to differentiation and contributes a majority of cells to the heart. *Dev. Cell* 5 877–889. 10.1016/s1534-5807(03)00363-0 14667410 PMC5578462

[B12] Christoffels V. M. Mommersteeg M. T. M. Trowe M. O. Prall O. W. J. De Gier-De Vries C. Soufan A. T. (2006b). Formation of the venous pole of the heart from an Nkx2-5-negative precursor population requires Tbx18. *Circ. Res.* 98 1555–1563. 10.1161/01.res.0000227571.84189.65 16709898

[B13] Christophersen I. E. Rienstra M. Roselli C. Yin X. Geelhoed B. Barnard J. (2017). Large-scale analyses of common and rare variants identify 12 new loci associated with atrial fibrillation. *Nat. Genet.* 49 946–952. 28416818 10.1038/ng.3843 PMC5585859

[B14] De Maziere A. M. van Ginneken A. C. Wilders R. Jongsma H. J. Bouman L. N. (1992). Spatial and functional relationship between myocytes and fibroblasts in the rabbit sinoatrial node. *J. Mol. Cell Cardiol.* 24 567–578. 10.1016/0022-2828(92)91041-3 1518074

[B15] Dixon J. R. Gorkin D. U. Ren B. (2016). Chromatin domains: the unit of chromosome organization. *Mol. Cell* 62 668–680. 10.1016/j.molcel.2016.05.018 27259200 PMC5371509

[B16] Espinoza-Lewis R. A. Liu H. Sun C. Chen C. Jiao K. Chen Y. (2011). Ectopic expression of Nkx2.5 suppresses the formation of the sinoatrial node in mice. *Dev. Biol* 356 359–369. 10.1016/j.ydbio.2011.05.663 21640717 PMC3143305

[B17] Espinoza-Lewis R. A. Yu L. He F. Liu H. Tang R. Shi J. (2009). Shox2 is essential for the differentiation of cardiac pacemaker cells by repressing Nkx2-5. *Dev. Biol.* 327 376–385. 10.1016/j.ydbio.2008.12.028 19166829 PMC2694185

[B18] Fernandez-Perez A. Sathe A. A. Bhakta M. Leggett K. Xing C. Munshi N. V. (2019). Hand2 selectively reorganizes chromatin accessibility to induce pacemaker-like transcriptional reprogramming. *Cell Rep.* 27 2354–2369e7. 31116981 10.1016/j.celrep.2019.04.077 PMC6657359

[B19] Galang G. Mandla R. Ruan H. Jung C. Sinha T. Stone N. R. (2020). ATAC-seq Reveals an Isl1 enhancer that regulates sinoatrial node development and function. *Circ. Res.* 127 1502–1518. 10.1161/circresaha.120.317145 33044128 PMC7720845

[B20] Goodyer W. R. Beyersdorf B. M. Paik D. T. Tian L. Li G. Buikema J. W. (2019). Transcriptomic profiling of the developing cardiac conduction system at single-cell resolution. *Circ. Res.* 125 379–397. 10.1161/circresaha.118.314578 31284824 PMC6675655

[B21] Hanssen L. L. P. Kassouf M. T. Oudelaar A. M. Biggs D. Preece C. Downes D. J. (2017). Tissue-specific CTCF-cohesin-mediated chromatin architecture delimits enhancer interactions and function in vivo. *Nat. Cell Biol.* 19 952–961. 10.1038/ncb3573 28737770 PMC5540176

[B22] Hoogaars W. M. Engel A. Brons J. F. Verkerk A. O. de Lange F. J. Wong L. Y. (2007). Tbx3 controls the sinoatrial node gene program and imposes pacemaker function on the atria. *Genes Dev.* 21 1098–1112. 10.1101/gad.416007 17473172 PMC1855235

[B23] Hoogaars W. M. Tessari A. Moorman A. F. de Boer P. A. Hagoort J. Soufan A. T. (2004). The transcriptional repressor Tbx3 delineates the developing central conduction system of the heart. *Cardiovasc. Res.* 62 489–499. 10.1016/j.cardiores.2004.01.030 15158141

[B24] Joyner R. W. van Capelle F. J. (1986). Propagation through electrically coupled cells. How a small SA node drives a large atrium. *Biophys. J.* 50 1157–1164. 10.1016/s0006-3495(86)83559-7 3801575 PMC1329789

[B25] Kapoor N. Galang G. Marban E. Cho H. C. (2011). Transcriptional suppression of connexin43 by TBX18 undermines cell-cell electrical coupling in postnatal cardiomyocytes. *J. Biol. Chem.* 286 14073–14079. 10.1074/jbc.m110.185298 21205823 PMC3077608

[B26] Kapoor N. Liang W. Marban E. Cho H. C. (2013). Direct conversion of quiescent cardiomyocytes to pacemaker cells by expression of Tbx18. *Nat. Biotechnol.* 31 54–62. 10.1038/nbt.2465 23242162 PMC3775583

[B27] Kilpeläinen T. O. (2016). Genome-wide association studies and resting heart rate. *J. Electrocardiol.* 49 860–863. 10.1016/j.jelectrocard.2016.07.022 27519143

[B28] Kuratomi S. Ohmori Y. Ito M. Shimazaki K. Muramatsu S. Mizukami H. (2009). The cardiac pacemaker-specific channel Hcn4 is a direct transcriptional target of MEF2. *Cardiovasc. Res.* 83 682–687. 10.1093/cvr/cvp171 19477969

[B29] Lakatta E. G. Maltsev V. A. Vinogradova T. M. (2010). A coupled SYSTEM of intracellular Ca2+ clocks and surface membrane voltage clocks controls the timekeeping mechanism of the heart’s pacemaker. *Circ. Res.* 106 659–673. 10.1161/circresaha.109.206078 20203315 PMC2837285

[B30] Larson E. D. Marsh A. J. Harrison M. M. (2021). Pioneering the developmental frontier. *Mol. Cell* 81 1640–1650. 10.1016/j.molcel.2021.02.020 33689750 PMC8052302

[B31] Li H. Li D. Wang Y. Huang Z. Xu J. Yang T. (2019). Nkx2-5 defines a subpopulation of pacemaker cells and is essential for the physiological function of the sinoatrial node in mice. *Dev.* 146:dev178145. 10.1242/dev.178145 PMC6679370 31320323

[B32] Liang X. Zhang Q. Cattaneo P. Zhuang S. Gong X. Spann N. J. (2015). Transcription factor ISL1 is essential for pacemaker development and function. *J. Clin. Invest.* 125 3256–3268. 10.1172/jci68257 26193633 PMC4563735

[B33] Liu C. Liu W. Palie J. Lu M. F. Brown N. A. Martin J. F. (2002). Pitx2c patterns anterior myocardium and aortic arch vessels and is required for local cell movement into atrioventricular cushions. *Development* 129 5081–5091. 10.1242/dev.129.21.5081 12397115

[B34] Mifsud B. Tavares-Cadete F. Young A. N. Sugar R. Schoenfelder S. Ferreira L. (2015). Mapping long-range promoter contacts in human cells with high-resolution capture Hi-C. *Nat. Genet.* 47 598–606. 10.1038/ng.3286 25938943

[B35] Molkentin J. D. Lin Q. Duncan S. A. Olson E. N. (1997). Requirement of the transcription factor GATA4 for heart tube formation and ventral morphogenesis. *Genes Dev.* 11 1061–1072. 10.1101/gad.11.8.1061 9136933

[B36] Mommersteeg M. T. Hoogaars W. M. Prall O. W. de Gier-de Vries C. Wiese C. Clout D. E. (2007b). Molecular pathway for the localized formation of the sinoatrial node. *Circ. Res.* 100 354–362. 10.1161/01.res.0000258019.74591.b3 17234970

[B37] Mommersteeg M. T. M. Brown N. A. Prall O. W. J. De Gier-De Vries C. Harvey R. P. Moorman A. F. M. (2007a). Pitx2c and Nkx2-5 are required for the formation and identity of the pulmonary myocardium. *Circ. Res.* 101 902–909. 10.1161/circresaha.107.161182 17823370

[B38] Montefiori L. E. Sobreira D. R. Sakabe N. J. Aneas I. Joslin A. C. Hansen G. T. (2018). A promoter interaction map for cardiovascular disease genetics. *e*L*ife* 7:e35788. 10.7554/eLife.35788 PMC6053306 29988018

[B39] Moskowitz I. P. Kim J. B. Moore M. L. Wolf C. M. Peterson M. A. Shendure J. (2007). A molecular pathway including Id2, Tbx5, and Nkx2-5 required for cardiac conduction system development. *Cell* 129 1365–1376. 10.1016/j.cell.2007.04.036 17604724

[B40] Nikolaidou T. Aslanidi O. V. Zhang H. I Efimov R. (2012). Structure-function relationship in the sinus and atrioventricular nodes. *Pediatr. Cardiol.* 33 890–899. 10.1007/s00246-012-0249-0 22391764 PMC3703519

[B41] Opthof T. de Jonge B. Mackaay A. J. Bleeker W. K. Masson-Pevet M. Jongsma H. J. (1985). Functional and morphological organization of the guinea-pig sinoatrial node compared with the rabbit sinoatrial node. *J. Mol. Cell Cardiol.* 17 549–564. 10.1016/s0022-2828(85)80024-9 4020878

[B42] Pashmforoush M. Lu J. T. Chen H. Amand T. S. Kondo R. Pradervand S. (2004). Nkx2-5 pathways and congenital heart disease; loss of ventricular myocyte lineage specification leads to progressive cardiomyopathy and complete heart block. *Cell* 117 373–386. 15109497 10.1016/s0092-8674(04)00405-2

[B43] Ramirez J. Duijvenboden S. V. Ntalla I. Mifsud B. Warren H. R. Tzanis E. (2018). Thirty loci identified for heart rate response to exercise and recovery implicate autonomic nervous system. *Nat. Commun.* 9:1947. 10.1038/s41467-018-04148-1 PMC5955978 29769521

[B44] Ren J. Han P. Ma X. Farah E. N. Bloomekatz J. Zeng X. I. (2019). Canonical Wnt5b signaling directs outlying Nkx2.5+ mesoderm into pacemaker cardiomyocytes. *Dev. Cell* 50 729–743.e5. 31402282 10.1016/j.devcel.2019.07.014 PMC6759400

[B45] Roselli C. Chaffin M. D. Weng L. C. Aeschbacher S. Ahlberg G. Albert C. M. (2018). Multi-ethnic genome-wide association study for atrial fibrillation. *Nat. Genet.* 50 1225–1233. 29892015 10.1038/s41588-018-0133-9 PMC6136836

[B46] Sati S. Cavalli G. (2017). Chromosome conformation capture technologies and their impact in understanding genome function. *Chromosoma* 126 33–44. 10.1007/s00412-016-0593-6 27130552

[B47] Schoenfelder S. Furlan-Magaril M. Mifsud B. Tavares-Cadete F. Sugar R. Javierre B. M. (2015). The pluripotent regulatory circuitry connecting promoters to their long-range interacting elements. *Genome Res.* 25 582–597. 10.1101/gr.185272.114 25752748 PMC4381529

[B48] Sun Y. Liang X. Najafi N. Cass M. Lin L. Cai C. L. (2007). Islet 1 is expressed in distinct cardiovascular lineages, including pacemaker and coronary vascular cells. *Dev. Biol.* 304 286–296. 10.1016/j.ydbio.2006.12.048 17258700 PMC2582044

[B49] Tessadori F. van Weerd J. H. Burkhard S. B. Verkerk A. O. de Pater E. Boukens B. J. (2012). Identification and functional characterization of cardiac pacemaker cells in zebrafish. *PLoS One* 7:e47644. 10.1371/journal.pone.0047644 23077655 PMC3473062

[B50] Thomas K. Henley T. Rossi S. Costello M. J. Polacheck W. Griffith B. E. (2021). Adherens junction engagement regulates functional patterning of the cardiac pacemaker cell lineage. *Dev. Cell* 56 1498–1511e7.^∗^1498-1511 e7 33891897 10.1016/j.devcel.2021.04.004 PMC8137639

[B51] van Eif V. W. Protze S. Bosada F. M. Yuan X. Sinha T. van Duijvenboden K. (2020). Genome-wide analysis identifies an essential human TBX3 pacemaker enhancer. *Circ. Res.* 127 1522–1535. 10.1161/circresaha.120.317054 33040635 PMC8153223

[B52] van Eif V. W. W. Stefanovic S. van Duijvenboden K. Bakker M. Wakker V. de Gier-de Vries C. (2019). Transcriptome analysis of mouse and human sinoatrial node cells reveals a conserved genetic program. *Development* 146:dev173161. 10.1242/dev.173161 30936179

[B53] van Steensel B. Belmont A. S. (2017). Lamina-associated domains: links with chromosome architecture, heterochromatin, and gene repression. *Cell* 169 780–791. 10.1016/j.cell.2017.04.022 28525751 PMC5532494

[B54] Vedantham V. Evangelista M. Huang Y. Srivastava D. (2013). Spatiotemporal regulation of an Hcn4 enhancer defines a role for Mef2c and HDACs in cardiac electrical patterning. *Dev. Biol.* 373 149–162. 10.1016/j.ydbio.2012.10.017 23085412 PMC3510001

[B55] Vedantham V. Galang G. Evangelista M. Deo R. C. Srivastava D. (2015). RNA sequencing of mouse sinoatrial node reveals an upstream regulatory role for Islet-1 in cardiac pacemaker cells. *Circ. Res.* 116 797–803. 10.1161/circresaha.116.305913 25623957 PMC4344860

[B56] Weinberger F. Mehrkens D. Friedrich F. W. Stubbendorff M. Hua X. Muller J. C. (2012). Localization of Islet-1-positive cells in the healthy and infarcted adult murine heart. *Circ. Res.* 110 1303–1310. 10.1161/circresaha.111.259630 22427341 PMC5559221

[B57] Wiese C. Grieskamp T. Airik R. Mommersteeg M. T. Gardiwal A. de Gier-de Vries C. (2009). Formation of the sinus node head and differentiation of sinus node myocardium are independently regulated by Tbx18 and Tbx3. *Circ. Res.* 104 388–397. 10.1161/circresaha.108.187062 19096026

[B58] Ye W. Wang J. Song Y. Yu D. Sun C. Liu C. (2015). A common Shox2-Nkx2-5 antagonistic mechanism primes the pacemaker cell fate in the pulmonary vein myocardium and sinoatrial node. *Development* 142 2521–2532. 26138475 10.1242/dev.120220 PMC4510860

[B59] Zhang M. Hill M. C. Kadow Z. A. Suh J. H. Tucker N. R. Hall A. W. (2019). Long-range Pitx2c enhancer-promoter interactions prevent predisposition to atrial fibrillation. *Proc. Natl. Acad. Sci. U.S.A.* 116 22692–22698. 10.1073/pnas.1907418116 31636200 PMC6842642

